# Acute Haemolytic Anaemia Secondary to Lead Poisoning: A Case Report

**DOI:** 10.7759/cureus.103043

**Published:** 2026-02-05

**Authors:** Darshdeep Kaur

**Affiliations:** 1 Intensive Care Unit, Queen Elizabeth II (QEII) Hospital, Brisbane, AUS

**Keywords:** basophilic stippling, blood lead, blood lead levels, haemolytic anaemia, lead, lead toxicity

## Abstract

Lead toxicity (plumbumism) is a rare but clinically significant diagnosis in developed countries. It can manifest with a wide array of systemic and haematological symptoms. Due to its non-specific presentation and overlap with common conditions such as iron deficiency anaemia, diagnosis may be delayed without a high index of suspicion. We report a case of a 30-year-old previously healthy male patient who presented with generalised abdominal pain, jaundice, constipation, and lethargy. Laboratory investigations revealed a microcytic, hypochromic anaemia with indirect hyperbilirubinaemia and Coombs-negative haemolysis. Peripheral smear demonstrated basophilic stippling, prompting a lead level assessment, which returned markedly elevated (73.3 µg/dL). Upon further history, the patient disclosed recent ingestion of Indian Ayurvedic medicine, which was identified as the likely source of lead exposure. Chelation therapy was initiated with clinical improvement. This case highlights the importance of considering lead toxicity in the differential diagnosis of haemolytic anaemia, especially in patients using complementary or imported medicines. Peripheral smear findings, particularly basophilic stippling, can provide a vital clue. Early recognition and chelation therapy are essential to prevent long-term complications. Clinicians should maintain caution for lead poisoning in atypical anaemia presentations and take thorough exposure histories, including the use of traditional medicines.

## Introduction

Lead, or plumbum, is a ubiquitous element that affects almost all physiological systems, as there is no safe amount of lead within the body [1]. Exposure can occur through occupational, environmental, or accidental sources, e.g., lead-based paint in older homes, contaminated soil, or lead in water pipes. Lead toxicity, or plumbumism, can be a significant public health concern, particularly in industrialized and developing regions [2].

Clinically, patients with lead poisoning can present with a diverse spectrum of signs and symptoms, including abdominal pain, nausea, vomiting, fatigue, and cognitive dysfunction [3]. The severity of these symptoms is dependent on the degree of exposure and the blood level concentration of lead. Of particular concern, especially within this case, are the haematological effects, notably microcytic, hypochromic anaemia due to impaired haem synthesis and shortened erythrocyte lifespan [2]. The clinical presentation of lead-induced anaemia often mimics that of iron deficiency anaemia, complicating diagnosis and management. Basophilic stippling of red cells and elevated free erythrocyte protoporphyrin are key laboratory findings found within plumbumism [4-6]. Prompt identification of these features is crucial for early diagnosis and intervention to prevent irreversible organ damage.

## Case presentation

A 30 year-old male patient, without any underlying medical conditions, presented to his general practitioner with a five-day history of generalised mild abdominal pain, constipation, jaundice, lethargy, nausea, and vomiting. Upon examination, scleral icterus and generalised abdominal tenderness were noted; however, there was no organomegaly or features of peritonism. Full blood count and general blood chemistry revealed an acute anaemia with hyperbilirubinaemia. He was then referred to his local emergency department, where he was admitted for further investigation.

**Figure 1:**
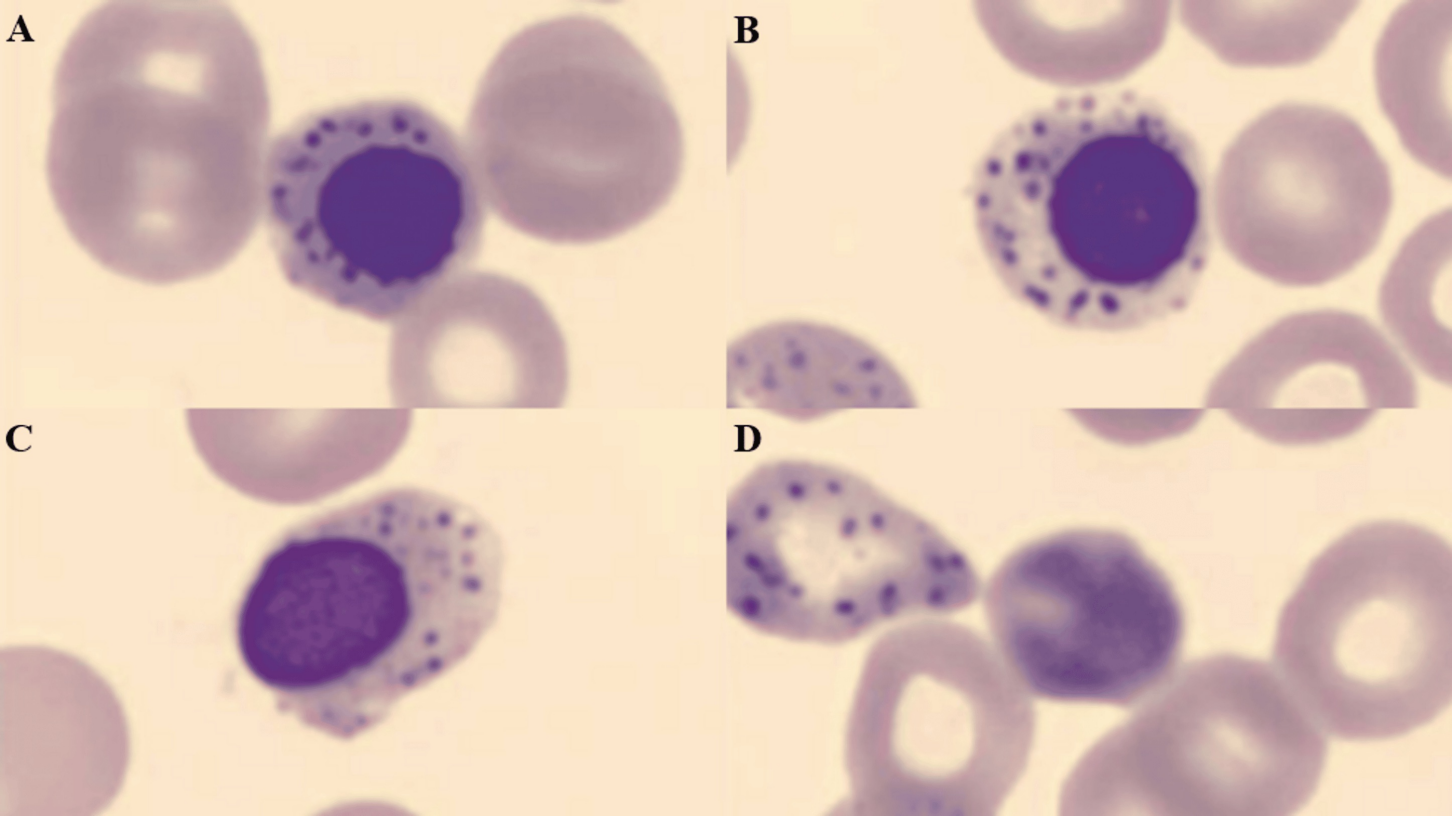
(A-D) Peripheral blood smear showing coarse basophilic stippling seen in red blood cells and nucleated red blood cells.

**Figure 2:**
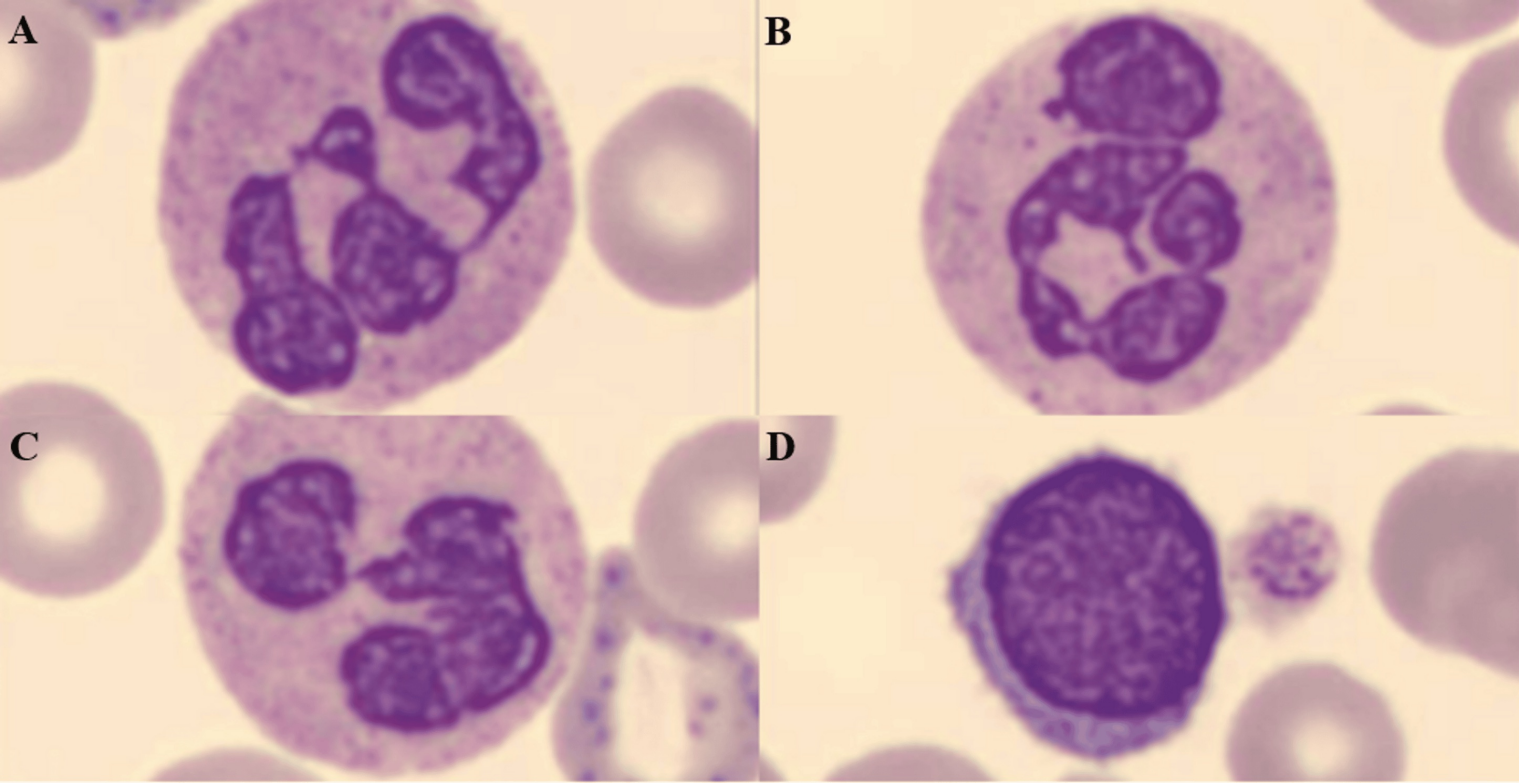
(A-D) Peripheral blood smear showing basophilic stippling in white blood cells as well.

Repeat laboratory tests confirmed an acute microcytic, hypochromic anaemia without clinical features of acute bleeding (see Table 1 for values). Hyperbilirubinaemia, marked reticulocytosis, and a negative Coombs test were also present, confirming an acute haemolytic anaemia along with other laboratory tests, including an increased lactate dehydrogenase (LDH) and decreased haptoglobin. Peripheral blood smear demonstrated basophilic stippling and anisopoikilocytosis (see Figure 1 for basophilic stippling). Basophilic stippling was also seen in white blood cells (see Figure 2). An abdominal ultrasound confirmed no biliary obstructions, hepatomegaly, or hepatic lesion but revealed a mild hepatosteatosis.

**Table 1:** Laboratory results upon initial presentation. MCV: mean corpuscular volume; MCH: mean corpuscular haemoglobin; AHG: anti-human globulin; LDH: lactate dehydrogenase

	Value	Reference range
Haemoglobin	85 g/L	135-189 g/L
MCV	74 fL	80-100 fL
MCH	25.6 pg	27-33 pg
Red cell count	3.32 × 10^12^/L	4.5-6 × 10^12^/L
Reticulocytes	153 × 10^9^/L	10-100 × 10^9^/L
Haptoglobin	0.25 g/L	0.40-2.80 g/L
Direct AHG (Coombs test)	Negative	
Bilirubin	43 µmol/L	<20 µmol/L
LDH	283 U/L	120-250 U/L

Although lead toxicity is rare in Australia, a recommendation of lead testing was made by pathology, given the peripheral blood film findings and clinical features prompting presentation. The blood lead level was significantly elevated at 73.3 µg/dL (normal <5.0 µg/dL). Upon further history, the patient revealed he had been taking Indian ayurvedic medications (unknown name and dose) intermittently over the last two months to aid with lethargy and fertility, with the last ingestion being two days prior to symptom onset.

Chelation therapy was commenced as per instructions from the local toxicology department. Succimer, or dimercaptosuccinic acid, oral capsules were commenced three times per day (600, 400, and 400 mg) for the first five days, then twice daily for two weeks. Follow-up lead levels were taken at the completion of chelation therapy and repeated three weeks later, which showed 34 µg/dL and undetectable lead levels, respectively. Unfortunately, repeat blood films were not completed to check haematological recovery.

## Discussion

This case underscores the clinical importance of recognising lead poisoning as a differential diagnosis in patients with haemolytic anaemia and abdominal symptoms, even in countries like Australia, where lead exposure is considered rare. The constellation of microcytic, hypochromic anaemia, basophilic stippling, and elevated blood lead levels is characteristic of plumbumism. Lead inhibits key enzymes in the haem biosynthesis pathway, including delta-aminolevulinic acid dehydratase (ALAD) and ferrochelatase, leading to defective haem production and accumulation of erythrocyte precursors [2,5]. Additionally, oxidative membrane damage contributes to the shortened lifespan of erythrocytes, culminating in haemolysis [4].

The findings in this patient-severe anaemia, reticulocytosis, indirect hyperbilirubinaemia, and Coombs-negative haemolysis-are classical for lead-induced haemolysis. Basophilic stippling, while often seen in lead toxicity, can mimic other rare haematological disorders, such as erythrocyte pyrimidine 5′-nucleotidase deficiency. A recent case study by Warang et al. highlighted a nearly identical diagnostic challenge in a patient with severe haemolytic anaemia and marked stippling, reinforcing the importance of lead level testing in such presentations [7].

Traditional remedies such as Ayurvedic medicines are well-documented sources of lead exposure, often contaminated during preparation or storage [8]. Ayurvedic medicines supplied within Australia are regulated by the Therapeutic Goods Administration (TGA) and are made in accordance with strict manufacturing practices and are, therefore, at a lower risk of exposure to lead or other heavy metals like mercury or arsenic [9]. However, production of Ayurvedic medications overseas does not have such strict regulations. This case aligns with growing literature on the need for clinicians to inquire specifically about complementary or alternative medicine use when evaluating unexplained systemic symptoms or haematological abnormalities.

Chelation therapy remains the cornerstone of treatment for patients with symptomatic or significant lead levels. Timely recognition and intervention are crucial, as chronic lead toxicity can result in irreversible neurological and renal impairment [10]. Ultimately, this case illustrates the importance of maintaining a high index of suspicion for lead toxicity, especially in atypical anaemia presentations, and highlights the diagnostic value of peripheral smear findings in guiding appropriate investigations.

Lead toxicity is a notifiable condition in Queensland, Australia. Therefore, under the Public Health Act 2005, all directors of pathology are required to notify the Chief Executive of Queensland Health of blood lead levels ≥ 5 µg/dL [11].

## Conclusions

This case illustrates the diagnostic challenge and clinical significance of lead toxicity, particularly in non-endemic settings such as Australia. The patient's presentation with abdominal symptoms, jaundice, and haemolytic anaemia initially suggested a broad differential, but careful examination of the peripheral blood smear-specifically the presence of basophilic stippling-prompted targeted lead level testing. The markedly elevated blood lead concentration, in the context of recent Ayurvedic medication use, confirms a preventable and potentially under-recognised source of heavy metal exposure. This highlights the critical role of detailed history-taking, including the use of complementary and alternative medicines, in uncovering hidden toxicological causes in addition to a detailed environmental and occupational history. Early diagnosis facilitated prompt chelation therapy, which is essential to prevent long-term complications such as nephropathy and neurocognitive decline. Clinicians must maintain a high index of suspicion for lead poisoning in atypical anaemia presentations and use accessible diagnostic tools like peripheral smears to guide further investigations. Lead toxicity, though uncommon in developed countries, should be considered in patients with unexplained microcytic anaemia, basophilic stippling, or haemolysis, especially when other common causes have been excluded. As this case demonstrates, awareness and timely intervention can significantly alter outcomes in patients with otherwise overlooked toxic exposures. This case also reinforces the need for clinician awareness of non-traditional sources of heavy metal exposure, including imported herbal supplements, and highlights the importance of interdisciplinary management involving toxicology and public health authorities.
